# KnvResGAT: SARS-CoV-2 sequence classification using k-mer natural vector and graph attention networks

**DOI:** 10.1186/s13104-026-07695-9

**Published:** 2026-02-12

**Authors:** Wenping Yu, Yongjie Deng, Zhewen Li, Wenbo Dong

**Affiliations:** College of Artificial Intelligence, Tianjin University of Science and Technology, Tianjin, China

**Keywords:** SARS-CoV-2, Sequence classification, k-mer natural vector, Graph neural network, Attention mechanism, Residual connection

## Abstract

**Objective:**

We propose KnvResGAT for efficient SARS-CoV-2 lineage classification by combining k-mer Natural Vector (KNV) representations with a residual multi-head Graph Attention Network (GAT) on a k-nearest-neighbor (kNN) similarity graph constructed in the KNV feature space.

**Results:**

On a time-aware per-lineage split of 182,851 curated SARS-CoV-2 genomes spanning 103 Pango lineages, KnvResGAT achieved 0.9729 accuracy and 0.9636 Macro-F1. Under the same split, it outperformed Pangolin (0.9673 accuracy, 0.9471 Macro-F1) and a strong deep baseline ResMLP (0.9654 accuracy, 0.9520 Macro-F1), demonstrating improved generalization for multi-class lineage classification.

## Introduction

The COVID-19 pandemic highlights the need for accurate viral genome classification, which is critical for understanding evolution, transmission, and host adaptation [1–3]. Coronaviruses are complex single-stranded RNA viruses with high mutation rates and cross-species transmission potential [2]. Studies indicate SARS-CoV-2 likely originated in animals and jumped to humans via intermediate hosts [3]. Efficient genome analysis methods are essential for surveillance, vaccine development, and epidemic control.

Beyond SARS-CoV-2, globalization, urbanization, and climate change are reshaping the geographic risk landscape for vector-borne viral diseases and their cross-border spread. Recent reviews and regional surveillance studies on Aedes-associated arboviruses and environmental drivers further underscore the value of scalable molecular surveillance pipelines for timely detection and response [4–9].

Traditional classification using phylogenetic trees and multiple sequence alignment (MSA) is effective but computationally intensive and error-prone on large viral datasets [10]. MSA also struggles with highly variable RNA viruses due to frequent mutation and recombination [11]. Consequently, machine learning and deep learning approaches have been applied, offering strong generalization for genome classification [12].

Graph neural networks (GNNs) can capture complex relationships in non-Euclidean data through message passing. GNNs with attention mechanisms can adaptively weight neighborhood information in the graph, which may improve classification performance and training stability [13, 14].

Based on this background, we introduce KnvResGAT, which integrates k-mer natural vectors with a graph attention network and residual connections. Our approach consists of three components:

1. Graph construction from k-mer natural vectors: Each sequence is encoded into a k-mer natural vector capturing local statistics and global distribution. We construct a k-nearest-neighbor (kNN) graph in this feature space, where nodes represent sequences and edges connect local neighborhoods to capture higher-order associations.
2. GNN with attention and residual connections: On the kNN graph, we employ a multi-head graph attention network with residual connections to support stable feature propagation and effective neighborhood aggregation for classification.
3. Adaptive attention-based feature emphasis: A multi-head attention mechanism enables KnvResGAT to adaptively weight neighbor contributions and emphasize informative patterns in sequence-derived features. We describe this as an attention-based aggregation strategy rather than a direct biological interpretation of specific genomic regions.

Our contributions are: (1) a kNN-based graph representation of SARS-CoV-2 sequences using k-mer natural vectors, capturing local statistical features in a sequence-level similarity graph; (2) the KnvResGAT model, which integrates residual connections with graph attention to perform adaptive neighborhood aggregation for lineage classification; and (3) a comprehensive evaluation across k-mer settings and training-data regimes, including comparisons with GNN baselines and conventional classifiers under a shared experimental protocol.

## Related work

Initial virus classification relied on multiple sequence alignment (MSA) and phylogenetic methods, but these approaches become impractical for large and highly variable datasets [15]. As a result, alignment-free k-mer-based methods have gained popularity. Such methods decompose genomes into k-length subsequences and compute frequency, position, and moment statistics, efficiently capturing sequence patterns without alignment [16]. However, k-mer representations alone may miss higher-order and long-range patterns in distant evolutionary relationships [17, 18].

Position weight matrix (PWM) methods calculate position-specific nucleotide probabilities to enhance feature representation [19], but they depend on sequence alignment and do not scale well to massive unaligned datasets.

Minimizer-based methods generate compact embeddings for short-read data. For example, Kraken [19] and CoMeta [20] use k-mer indices for metagenomic classification, and probabilistic sequence signatures can accurately group reads [21]. These approaches are highly efficient and widely adopted in practical analysis pipelines, but they are primarily designed for short-read or tool-oriented classification and are more difficult to extend to large-scale, full-length viral genome modeling tasks.

Deep learning methods have also shown promise in sequence analysis. Convolutional and recurrent neural networks can automatically learn discriminative features from raw sequences [22]. Kernel-based approaches, such as the k-spectrum kernel and local alignment kernel, generate fixed-length representations for support vector machine (SVM) classification, but they often face scalability challenges when applied to large genomic datasets [23].

Graph-based approaches have recently attracted increasing attention. De Bruijn graphs combined with graph convolutional or attention networks have achieved success in DNA sequence analysis [24]. Hypergraph neural networks further extend this idea by modeling higher-order relationships among sequences [25]; for instance, Seq-HyGAN constructs a sequence hypergraph with multi-level attention to capture both local and global context [26]. These methods typically rely on explicit k-mer overlap structures or hypergraph formulations, whereas alternative graph constructions based on sequence-level similarity in feature space offer a complementary way to model relationships among complete genomes. In addition, one-hot encoding of spike proteins can perform well in specific scenarios [27], but k-mer–based representations generally capture richer sequence information [28].

Overall, existing methods span machine learning, deep learning, and graph-based models, each with distinct trade-offs. A key challenge remains how to efficiently integrate local statistical features, global structural information, and neighborhood-level dependencies within a unified framework. These considerations motivate our approach, which combines k-mer natural vector representations with graph attention mechanisms to achieve effective and scalable SARS-CoV-2 sequence classification.

## Method

**Fig. 1:**
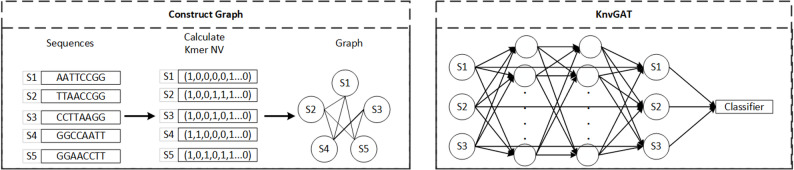
Model Architecture

We proposeKnvResGAT, combining k-mer NV sequence representation with a ResNet-style graph attention network (GAT). The workflow has three steps: k-mer NV construction, graph construction, and GAT-ResNet training. The overall process is illustrated in Fig. 1.

### k-mer natural vector

Each sequence is segmented by a sliding window of length k. For each k-mer, we compute its occurrence frequency f, average position p, and m-th order central moment µ. These three statistics form the k-mer natural vector. The vector has three parts of length 4 k: the counts, positions, and moments of all possible k-mers, producing a fixed-length representation.

$\:{\mathrm{n}}_{{\mathrm{l}}_{\mathrm{i}}}$ represents the occurrence frequency of the nucleotide sequence k-mer $\:{\mathrm{l}}_{\mathrm{i}}$ in sequence S.

$\:{{\upmu\:}}_{{\mathrm{l}}_{\mathrm{i}}}$ represents the average position at which the nucleotide sequence k-mer $\:{\mathrm{l}}_{\mathrm{i}}$ appears in S.

$\:{\mathrm{D}}_{\mathrm{m}}^{{\mathrm{l}}_{\mathrm{i}}}=\sum\:_{\mathrm{m}=1}^{{\mathrm{n}}_{{\mathrm{l}}_{\mathrm{i}}}}\frac{{\left({\mathrm{l}}_{\mathrm{i}}\left[\mathrm{j}\right]-{{\upmu\:}}_{{\mathrm{l}}_{\mathrm{i}}}\right)}^{\mathrm{m}}}{{\mathrm{n}}_{{\mathrm{l}}_{\mathrm{i}}}^{\mathrm{m}-1}{\left(\mathrm{n}-\mathrm{k}+1\right)}^{\mathrm{m}-1}},\mathrm{m}=1,2...{,\mathrm{n}}_{{\mathrm{l}}_{\mathrm{i}}}$ represents the m-th order central moment of the nucleotide sequence k-mer $\:{\mathrm{l}}_{\mathrm{i}}$ in S.

### Graph structure construction

After obtaining the k-mer natural vector representation for each viral sequence, we further organize these vectors into a graph structure to enable relational modeling with graph neural networks (GNNs). In this graph, each node corresponds to a viral sequence, and node features are given by the associated k-mer natural vector or its reduced-dimensional representation.

To characterize similarity between sequences, we compute pairwise distances in the feature space using the Euclidean metric. Instead of relying on a global distance threshold, we construct the graph using a k-nearest neighbor (kNN) strategy: for each node, edges are created to its k most similar neighboring sequences. This keeps the local neighborhood size fixed and yields a stable, well-connected topology across experimental settings.

In our implementation, feature normalization can be optionally applied prior to distance computation (when enabled, normalization is fitted on the training split and then applied consistently to validation and test splits). The kNN graph is symmetrized and treated as undirected; by construction, it contains no isolated nodes and each node has at least k neighbors. We store edge weights as a distance-to-similarity transformation so that closer neighbors receive larger weights, which can be used by baselines that support weighted edges; attention-based models primarily learn neighborhood importance through attention coefficients while sharing the same fixed graph structure for fair comparison.

### Neural network model

Each node’s initial feature ​ is the (possibly reduced) k-mer NV. KnvResGAT has three layers of graph attention convolution with multi-head attention. Layer 1 uses 4 heads (output dimension D′ each, concatenated to 4 D′); Layer 2 also uses 4 heads; Layer 3 uses 1 head to map features to the classification output. To mitigate vanishing gradients, we add a residual shortcut: after a linear mapping, the input feature is added to the third layer’s output. The network uses ELU activation, dropout, and a log-softmax layer for classification. $\:{\mathrm{x}}_{\mathrm{i}}{\mathrm{W}}_{\mathrm{r}}$.

## Experiments

### Dataset

We conducted experiments on a large-scale SARS-CoV-2 genome dataset curated from the GISAID EpiCoV database. Raw genome sequences and metadata were processed using a standardized pipeline to obtain a high-quality dataset for supervised lineage classification.

Starting from 229,849 merged records, we applied strict quality control. Sequences were retained only if they had genome lengths between 29,000 and 30,000 nucleotides, were sampled from human hosts, and contained no ambiguous nucleotides beyond the standard A/C/G/T alphabet. Records with invalid or missing collection-date metadata were removed. After quality filtering, lineage screening, and deduplication at the nucleotide-sequence level, the final dataset contained 182,851 unique genomes.

To ensure reliable multi-class evaluation, we retained 103 Pango lineages, each with at least 300 sequences. The dataset spans from late 2019 to mid-2022, covering multiple epidemic phases and major variant transitions.

To reflect realistic deployment scenarios, we adopted a time-aware per-lineage split. For each lineage, sequences were sorted chronologically and split into training, validation, and test sets with ratios of 80%/10%/10%, respectively. This strategy avoids information leakage from future samples and enforces temporal generalization. The final split consists of 146,279 training, 18,291 validation, and 18,281 test sequences, and is shared across all experiments and baselines.

### Parameter settings

All experiments follow a unified experimental protocol to ensure fair and reproducible comparison across model architectures, graph construction strategies, and baselines.

For graph-based models, sequence representations are first computed using k-mer Natural Vectors with a fixed k value (k = 6 in the main experiments). Based on these representations, a k-nearest-neighbor (kNN) graph is constructed, where each node corresponds to a viral genome and edges connect the k most similar sequences in feature space. Unless otherwise stated, we use the same graph structure for all GNN variants within a given comparison.

To evaluate model robustness, architecture ablation experiments are conducted on the mutual graph setting using five random seeds (seed = 0–4). This includes GAT, GCN, and their enhanced or residual variants, all trained under identical graph topology and optimization settings.

To isolate the effect of graph construction strategies, we further compare plain, weighted, per-class hybrid, and mutual graph variants using a fixed seed (seed = 0), while keeping the backbone architecture unchanged.

For baseline comparisons, ResMLP is trained with five random seeds under the same data split, while conventional MLP classifiers and the external tool Pangolin are evaluated using their standard deterministic settings.

### Results

We evaluate the proposed KnvResGAT and representative GNN baselines under a controlled setting to isolate the effect of model architecture. All models use KNV features with k = 6 and share the same mutual kNN graph construction, ensuring that performance differences are attributable to the learning architecture rather than graph topology. Each configuration is repeated over five random seeds (0–4) and we report mean ± standard deviation.

Table 1 summarizes the results. Overall, KnvResGAT (enh_gat) achieves the best performance across the main classification metrics, reaching 0.9729 accuracy and 0.9636 Macro-F1 with very small variance, indicating stable training and consistent generalization. The enhanced residual GCN variant (enh_gcn) performs competitively but remains slightly below KnvResGAT on both accuracy and Macro-F1. In contrast, the vanilla backbones (gcn, gat) show substantially lower Macro-F1, suggesting that the enhancement/residual design is important for robust multi-class lineage classification in this large-scale setting.

**Table 1:** Performance comparison on KNV (k = 6) with mutual kNN graph (5 seeds)

Model	Graph	Runs	Accuracy	Macro-F1	Macro-*P*	Macro-*R*	NLL	Brier	ECE
KnvResGAT (enh_gat)	Mutual	5	0.9729 ± 0.0006	0.9636 ± 0.0016	0.9598 ± 0.0026	0.9700 ± 0.0007	0.1983 ± 0.0037	0.0499 ± 0.0009	0.0599 ± 0.0011
enh_gcn	Mutual	5	0.9716 ± 0.0011	0.9618 ± 0.0013	0.9577 ± 0.0019	0.9689 ± 0.0020	0.2072 ± 0.0037	0.0527 ± 0.0015	0.0661 ± 0.0010
gcn	Mutual	5	0.9434 ± 0.0052	0.9205 ± 0.0084	0.9245 ± 0.0080	0.9241 ± 0.0094	0.2957 ± 0.0121	0.0933 ± 0.0069	0.0498 ± 0.0056
gat	Mutual	5	0.9246 ± 0.0010	0.8974 ± 0.0015	0.8962 ± 0.0022	0.9055 ± 0.0022	0.3572 ± 0.0137	0.1202 ± 0.0019	0.0178 ± 0.0016

Metrics reported as mean ± std over 5 runs. Lower is better for NLL/Brier/ECE

Beyond accuracy-style metrics, Table 1 also reports probabilistic quality indicators. KnvResGAT yields the lowest NLL and Brier score, reflecting improved predictive confidence and overall calibration quality compared to the baselines. Collectively, these results demonstrate that combining attention-based neighborhood aggregation with the enhanced/residual design provides consistent gains under a fixed graph construction protocol.

### Baseline comparison with conventional classifiers

To further clarify where the gains come from, we provide two complementary comparisons. First, we conduct a graph construction ablation to isolate the impact of different graph-building methods while keeping the backbone fixed. Second, we compare against non-graph baselines, including MLP/ResMLP trained directly on the same KNV features and the external lineage assignment tool Pangolin.

Table 2 shows that graph construction is a major factor for performance. Using the same backbone, the mutual graph consistently yields the strongest results, while simpler strategies (plain/weighted) lead to substantial degradation. The per-class hybrid graph remains competitive but is slightly inferior to mutual in both accuracy and Macro-F1.

**Table 2:** Graph construction ablation (seed = 0, KNV k = 6)

Backbone	Graph method	Accuracy	Macro-F1	NLL	Brier	ECE
enh_gat	Mutual	0.9729	0.9636	0.1983	0.0499	0.0599
enh_gat	Perclass_hybrid	0.9673	0.9581	0.2482	0.0605	0.0621
enh_gat	Plain	0.9224	0.8982	0.7986	0.1290	0.0222
enh_gat	Weighted	0.9164	0.8977	0.8484	0.1426	0.0451
gcn	Mutual	0.9434	0.9205	0.2957	0.0933	0.0498
gcn	Perclass_hybrid	0.8431	0.8431	1.0450	0.3490	0.2607
gcn	Plain	0.8067	0.6451	0.7758	0.2887	0.1144
gcn	Weighted	0.8285	0.7141	0.7226	0.2612	0.1128

Lower is better for NLL/Brier/ECE

Table 3 reports baseline results without graph modeling. ResMLP provides a strong non-graph deep baseline, but it remains below the proposed KnvResGAT (enh_gat) under the mutual graph setting, indicating that the improvements are not solely due to a deeper MLP-style architecture. Pangolin performs competitively as an external reference but is still lower than our best graph-based setting on Macro-F1 under this evaluation protocol.

**Table 3:** Baseline comparison without graph modeling (KNV k = 6)

Model	Runs	Accuracy	Macro-F1	Macro-*P*	Macro-*R*
ResMLP (resmlp_torch)	5	0.9654 ± 0.0014	0.9520 ± 0.0033	0.9467 ± 0.0036	0.9639 ± 0.0027
MLP (mlp_sklearn)	1	0.9642	0.9510	0.9501	0.9570
Pangolin	5	0.9673 ± 0.0000	0.9471 ± 0.0000	0.9731 ± 0.0000	0.9411 ± 0.0000

ResMLP is reported as mean ± std over 5 seeds; MLP_sklearn is a single-run result; Pangolin is reported as mean ± std over 5 runs

### Model interpretability (preliminary analysis)

To provide additional transparency into model behavior, we include a preliminary interpretability analysis focused on attention coefficients over graph neighborhoods and feature attribution over k-mer natural vector components. We inspect the distribution of attention weights across neighbors for correctly classified samples and summarize whether high-weight neighbors tend to share the same lineage label. In addition, we compute gradient-based saliency scores with respect to the input k-mer natural vector to identify k-mers that contribute most strongly to the predicted lineage.

These analyses are intended to support qualitative understanding of the model’s decision process rather than to claim direct biological causality. Where relevant, we discuss whether highly attributed k-mers are consistent with lineage-defining patterns reported in prior literature, and we emphasize that biological validation requires dedicated downstream analyses.

## Conclusion

We introduced KnvResGAT, which integrates k-mer Natural Vector sequence encoding with a residual graph attention network. Unlike learned embeddings such as kmer2 vec, k-mer Natural Vector yields fixed-size representations with simple, alignment-free feature construction.We also evaluated kmer2vec (k2v) embeddings at k = 6; however, on the expanded 103-lineage dataset the k2v-based setting showed noticeably weaker and less stable performance than KNV, suggesting that k2v representations degrade as class diversity and temporal heterogeneity increase.

In experiments across k = 6, KnvResGAT consistently achieved higher accuracy than kmer2 vec-based models, especially at larger k. This indicates that k-mer Natural Vectors capture lineage-discriminative sequence patterns in a compact form. The residual GAT facilitates stable feature propagation across layers and adaptive neighborhood aggregation. Overall, KnvResGAT provides a practical alignment-free option for SARS-CoV-2 sequence classification under our experimental settings. More broadly, scalable sequence classification models complement ongoing molecular surveillance efforts across diverse viral diseases influenced by environmental and ecological drivers [4–9].

## Limitations

Data quality and diversity: The GISAID dataset may contain sequencing errors or sampling biases, and covers only known lineages up to 2022. Undersampled variants or geographic biases could affect the model’s applicability.

Sample size and class imbalance: Although our curated dataset contains 182,851 genomes, some lineages have relatively few samples, which may limit performance on rare classes. Results on extremely small or imbalanced subsets remain to be tested.

Generalizability: KnvResGAT was trained and evaluated on SARS-CoV-2 genomes. Its effectiveness on other viruses or organisms is unproven and likely requires retraining or adaptation.

Computational cost: Very large k-mer values (beyond 6) lead to high-dimensional features and denser graphs, increasing memory and runtime requirements. There is a trade-off between k-mer length and computational feasibility.

Model assumptions: The kNN graph construction assumes that local neighborhoods in the feature space reflect meaningful lineage relationships. Performance and computational cost may vary with the chosen neighborhood size k, since larger k increases graph density and memory/time overhead. Although we adopt kNN = 15 as a default setting (based on sensitivity experiments), different datasets may require re-tuning k for an optimal trade-off.

## Data Availability

The analyzed data sets generated during the present study are available from the corresponding author on reasonable request. We gratefully acknowledge all data contributors of the GISAID EpiCoV database. In accordance with GISAID’s data access and usage policies, we do not redistribute raw sequences or accession lists; researchers with appropriate GISAID access can reproduce the dataset by following the documented filtering procedure.GISAID acknowledgment and compliance: We gratefully acknowledge the authors and submitting laboratories for generating and sharing SARS-CoV-2 sequences through GISAID. We accessed the data in accordance with GISAID’s terms, and we will provide accession identifiers to qualified readers upon request in line with the data usage policy.
